# Knowledge, Attitude, and the Practice in Nosocomial Infection Control Among Afghan Healthcare Workers in Herat Afghanistan: A Cross‐Sectional Study

**DOI:** 10.1002/hsr2.71780

**Published:** 2026-01-29

**Authors:** Mohammad Masudi, Enayatollah Ejaz, Mohammad Faisal Wardak, Ali Rahimi, Nasar Ahmad Shayan, Joseph Christian Obnial, Don Eliseo Lucero‐Prisno

**Affiliations:** ^1^ Department of Pediatrics, Faculty of Medicine Herat University Herat Afghanistan; ^2^ Department of Curative Medicine, Faculty of Medicine Jami University Herat Afghanistan; ^3^ Paraclinical Department, Faculty of Medicine Ghalib University Herat Afghanistan; ^4^ Research Center Ghalib University Herat Afghanistan; ^5^ Department of Epidemiology and Biostatistics Western University London‐On Canada; ^6^ National Coalition of Independent Scholars Battleboro Vermont USA; ^7^ Department of Global Health and Development, Faculty of Public Health and Policy London School of Hygiene and Tropical Medicine London UK; ^8^ Office for Research, Innovation and Extension Services Southern Leyte State University Sogod Southern Leyte Philippines; ^9^ Center for University Research, University of Makati Makati City Philippines

**Keywords:** afghanistan, attitudes, healthcare workers, Herat, knowledge, nosocomial infections, practices

## Abstract

**Background:**

Hospital‐acquired infections (HIAs) remain a major threat to patient and healthcare worker (HCW) safety globally, with higher burden in low‐resource settings. Despite moderate knowledge among HCWs in many low‐income contexts, actual adherence to infection prevention practices is often poor. In Afghanistan, reductions in trained personnel and recurrent shortages of infection prevention supplies have further weakened routine infection control capacity. Evidence from the country is limited and data from western Afghanistan are lacking. This study assessed knowledge, attitude, and practice (KAP) regarding nosocomial infection control among HCWs in Herat.

**Methods:**

A cross‐sectional study of 433 HCWs in 14 Herat health facilities used a self‐administered, validated Persian KAP questionnaire to assess KAP toward nosocomial infection control; data were analyzed with SPSS 27 using descriptive statistics, chi‐square tests, and logistic regression.

**Results:**

A total of 433 HCWs participated (mean age 27.35 ± 6.16 years; 52.9% female). The overall median KAP scores were 53.8%, 76.9%, and 32.3%, respectively, revealing a substantial knowledge–practice gap. Higher knowledge was significantly associated with older age, higher education, being a doctor, longer experience, and employment in private hospitals (*p* < 0.05). Positive attitude was more common among females, married participants, those with advanced education, longer working hours, and private sector staff (*p* < 0.05). Better practice was observed among single participants and those working ≤ 8 h per day (*p* = 0.024). Multivariate analysis showed that Bachelor's/MD degree, medical profession, and official or volunteer status independently predicted higher knowledge; female gender, lower economic status, and working in private hospitals predicted higher attitude; while shorter working hours predicted higher practice (*p* < 0.05).

**Conclusion:**

HCWs in Herat, Afghanistan, exhibited moderate knowledge and positive attitudes toward nosocomial infection prevention, but adherence to infection control practices was low. Professional role, education, experience, training, and workload significantly influenced KAP, highlighting the combined impact of individual competencies and institutional factors. Strengthening infection control requires coordinated policies, adequate resources, hands‐on training, and workload management to bridge the knowledge–practice gap, reduce nosocomial infections, and improve patient safety.

## 1 Introduction

Nosocomial or hospital‐acquired infections (HAIs) are infections that appear 48–72 h after hospital admission or within 10 days of discharge [1, 2, 3]. Patients and healthcare workers (HCWs) alike are at risk, with HCWs particularly vulnerable during specimen handling, waste disposal, and equipment use [1, 4]. Immunocompromised individuals, the elderly, and children are especially susceptible. HAIs significantly compromise healthcare quality and safety [1, 3]. The Centers for Disease Control and Prevention (CDC) estimates approximately 37 000 deaths annually in the United States and 111 000 in Europe attributable to HAIs, adding about 16 million hospital days each year and imposing major economic burdens [5]. The most common types of HAIs include surgical site infections (19.6%), respiratory tract infections such as pneumonia (19.4%), urinary tract infections (19.0%), bloodstream infections (10.7%), and gastrointestinal infections (7.7%), with *Clostridioides difficile* responsible for nearly half of the latter [6]. Among the causative microorganisms, *Escherichia coli* (15.9%) and *Staphylococcus aureus* (12.3%) are the most frequently identified pathogens, followed by *Enterococcus species* (9.6%), *Pseudomonas aeruginosa* (8.9%), *Klebsiella species* (8.7%), coagulase‐negative *Staphylococci* (7.5%), *Candida species* (6.1%), *C. difficile* (5.4%), *Enterobacter species* (4.2%), *Proteus species* (3.8%), and *Acinetobacter species* (3.6%) [6, 7, 8]. Approximately 20% of these pathogens exhibit multidrug resistance, posing major therapeutic challenges. Although less common, viral agents such as *hepatitis B, hepatitis C*, and *HIV* account for 1%–5% of HAIs, primarily linked to unsafe injection and needle practices [9]. Globally, HAI prevalence ranges from 5.7% to 19.2% [10], with low‐ and middle‐income countries (15.5%) more affected than high‐income countries (7.6%) [11]. In high‐income settings such as Europe and the United States, prevalence is below 1%, whereas in Asia, Latin America, and sub‐Saharan Africa it can exceed 40% [12].

HCWs play a decisive role in preventing HAIs. A KAP approach helps identify behavioral and knowledge gaps that contribute to infections such as *methicillin‐resistant Staphylococcus aureus* (one of the leading causes of HAIs) and other common HAIs [13]. Assessing HCWs' understanding of infection control, antibiotic use, and transmission mechanisms is vital for designing effective interventions. Inadequate knowledge or poor attitudes can lead to unsafe practices, delayed diagnosis, and increased transmission risk. Studies have shown that physicians, nurses, dentists, and laboratory personnel often contribute to HAI spread due to lapses in aseptic technique [3, 14, 15].

KAP Studies from low‐ and middle‐income countries (LMICs) consistently report moderate‐to‐high knowledge levels (60%–90%) among HCWs but suboptimal practice scores (ranging from 48% in Ethiopia to 57% in India) [1, 16, 17, 18, 19]. This pattern highlights a persistent knowledge–practice gap, underscoring the need for context‐specific assessments to guide effective infection prevention strategies.

Afghanistan, a low income setting, has experienced substantial reduction in the operational capacity of public healthcare services since the political transition in 2021 [20]. Ongoing restrictions on female education and reduced availability of trained personnel have affected the replenishment of the clinical workforce pipeline, particularly in nursing and midwifery professions [21, 22]. These cadres are essential for routine infection prevention and control activities. Conflict‐affected healthcare facilities, including hospitals in Afghanistan, face recurrent shortages of basic Infection Prevention and Control (IPC) supplies such as Personal Protective Equipment (PPE) and hand hygiene and cleaning materials. Overcrowded wards, limited isolation capacity, damaged infrastructure, and poor Water Sanitation and Hygiene (WASH) services further hinder the implementation of routine IPC standards. Staff shortages, high turnover among cleaning personnel, low levels of IPC‐specific knowledge, inconsistent monitoring, and reliance on untrained volunteers exacerbate these challenges and reduce adherence to IPC practices. Together with supply chain disruptions and volatile security conditions, these factors increase the risk of HAIs in conflict‐affected contexts such as Afghanistan [23, 24, 25]. Empirical data from Afghanistan remain limited however. Only one study, conducted in a private hospital in Kabul, found that 22.2% of nurses had poor knowledge, 70.9% intermediate, and 6.8% good knowledge about HAI prevention [26]. Given the scarcity of data, particularly in western Afghanistan, this study addresses the need for research on HCWs' KAP regarding nosocomial infection control. Herat Province, one of Afghanistan's largest and most important healthcare hubs, lacks prior studies on this subject. Therefore, this study aims to assess the KAP of HCWs concerning nosocomial infections and their prevention. It also seeks to identify factors influencing these aspects to inform effective infection control strategies and contribute to a broader understanding of infection prevention challenges within Afghanistan's healthcare system and beyond.

## 2 Materials and Methods

### 2.1 Study Design and Setting

This cross‐sectional study was conducted among HCWs in Herat, Afghanistan, between October and December 2023. Fourteen health facilities were included (one regional teaching hospital and 13 private hospitals/clinics). These facilities were selected based on accessibility and convenience.

### 2.2 Study Participants

The inclusion criteria comprised HCWs who were medical doctors, nurses, midwives, dentists, laboratory technicians, or anesthetists and who were actively working in the selected health facilities during the study period, including those employed as interns, volunteers, official, or contract staff. The exclusion criteria included part‐time staff and administrative personnel. There was no minimum duration of employment required for participation.

### 2.3 Sampling and Recruitment

Convenience sampling was used. All HCWs who were present in the wards at the time of data collection were invited to participate. Information was communicated by the chief medical doctor, general supervisor, and heads of units. One questionnaire was provided to each participant and it was self‐administered in the presence of a researcher to allow clarification if needed and to ensure that each HCW completed only one questionnaire. On average, participants required approximately 10 min to complete the questionnaire.

### 2.4 Sample Size Determination

The sample size was calculated using a single population proportion formula with the following assumptions: a 50% expected proportion (*p* = 0.50), 5% margin of error (*d* = 0.05), and 95% confidence interval (*Z* = 1.96). An additional 10% was added to account for possible non‐response, increasing the required minimum sample size to 424 respondents. Ultimately, 433 completed questionnaires were obtained.

### 2.5 Data Collection and Instrument

Data were collected using a structured, self‐administered, closed‐ended questionnaire. A validated Persian version of a standard KAP instrument was used as the base tool, supplemented with items drawn from previously published KAP studies in this field [27, 28, 29, 30]. Content validity of the adapted instrument was evaluated by three experienced doctors. A pilot study was then conducted among 30 HCWs to assess reliability. Cronbach's alpha values for the final instrument were 0.78 for knowledge, 0.72 for attitude, and 0.86 for practice. All Cronbach alpha values were above the accepted minimum 0.70 which indicates good internal consistency.

### 2.6 Questionnaire and Scoring System

The questionnaire was comprised of four sections: (1) a sociodemographic questionnaire (12 items), (2) a knowledge questionnaire (13 items), (3) an attitude questionnaire (13 items), and (4) a practice questionnaire (13 items). The scoring system for the knowledge sections involved four responses, with 0 assigned to incorrect or inappropriate responses and 1 assigned to correct or appropriate responses, which yielded a range of 0‐13. The attitude section employed a three‐point scale (Agree = 1, Disagree = 0, and No Idea = 0), resulting in a score range of 0‐13. Additionally, practice sections that involved five response options (Always = 5, Mostly = 4, Sometimes = 3, Rarely = 2, and Never = 1) resulted in a score range of 13–65. The total KAP scores were divided into two groups, High and Low, using the median as the threshold. This method is commonly applied in studies where validated cut‐offs are not available and when score distributions are not normal [31]. For example, Masudi et al. (2025) used the median to categorize overall knowledge and attitude scores in their TB KAP study [32].

### 2.7 Data Analysis

The collected data were thoroughly checked for completeness and errors before the analysis. Incomplete questionnaires were excluded and treated as nonresponses. All completed questionnaires were entered into SPSS version 27 for analysis. The normal distribution of the continuous variables was assessed using established methods. The study variables were summarized using frequencies, proportions, medians, and interquartile ranges (IQR), particularly for quantitative variables that did not follow a normal distribution. Bivariate analysis was conducted using the chi‐squared test. For multivariate analysis, the KAP scores were dichotomized into two groups: High KAP (scores ≥ median) and Low KAP (scores < median). Unconditional binary logistic regression was used to identify independent variables associated with high KAP. Variables with missing values within completed questionnaires were handled using listwise deletion in regression analyses. Odds ratios with a 95% confidence level were computed, and a *p*‐value < 0.05 was considered statistically significant.

### 2.8 Ethical Considerations

The study was approved by the Institutional Review Board of Jami University on November 4, 2023 (protocol number 33). All participants provided written informed consent prior to participating in the study. The confidentiality and privacy of the participants were protected throughout the study, following the Declaration of Helsinki and the ethical principles of research involving human subjects.

## 3 Results

This study included 433 participants. The mean (SD) and the median age of participants were 27.35 (±6.157) and 26.0 years, respectively. Participants' gender distribution, marital status, hospital distribution, education level, profession, hours of work per day, number of patients in contact with, years of experience, and economic condition were analyzed.

Age group 25–29 years constituted 35.8% (*n* = 155) of participants and those aged 18–24 years accounted for 34.6% (*n* = 150). Females slightly outnumbered males with 52.9% (*n* = 229) versus 47.1% (*n* = 204), and 52.2% (*n* = 226) of participants were married. Regarding educational attainment, 49.7% (*n* = 215) were diploma holders, while 40.2% (*n* = 174) had a Bachelor's or MD degree. Nurses represented the largest professional category with 30.7% (*n* = 133). Herat Regional Hospital contributed the highest proportion of participants at 26.6% (*n* = 115). Most of the respondents (90.1%, *n* = 390) worked ≤ 8 h per day, and 50.1% (*n* = 217) reported contact with ≤ 23 patients per day. The majority (57.3%, *n* = 248) had ≤ 3 years of experience, and 46.2% (*n* = 200) were in the “average” economic status category (Table 1).

**Table 1:** The sociodemographic status of participants (Herat, Afghanistan, 2024).

		*N*	%
Age	18–24	150	34.6
	25–29	155	35.8
	30–34	78	18.0
	> = 35	50	11.5
Gender	Male	204	47.1
	Female	229	52.9
Marital status	Single	207	47.8
	Married	226	52.2
Education	Diploma	215	49.7
	Bachelor & MD	174	40.2
	Master & PhD	44	10.2
Professions	Medical doctor (MD)	101	23.3
	Nurse	133	30.7
	Midwives	76	17.6
	Medical technologists	67	15.5
	Others	56	12.9
Economic status	Good	107	24.7
	Average	200	46.2
	Poor	126	29.1
Employment status	Official	110	25.4
	Contract	179	41.3
	Volunteer	105	24.2
	Intern	39	9.0
Work shift	Morning	166	38.3
	Evening	111	25.6
	Nightly	58	13.4
	Circulating	98	22.6
Work hours (per day)	8 h or less	390	90.1
	More than 8 h	43	9.9
Number of patients visited (per day)	23 patients or less	217	50.1
	More than 23 patients	216	49.9
Experience (years)	3 years or less	248	57.3
	More than 3 years	185	42.7
Health centers	Herat regional hospital	115	26.6
	Loqman Hakim hospital	56	12.9
	Aria Apollo hospital	52	12.0
	Mehraban hospital	19	4.4
	Afghan Aria hospital	23	5.3
	Herat heart hospital	16	3.7
	Mofeed hospitals	19	4.4
	Afghan Salamat hospital	27	6.2
	Obaidi hospital	14	3.2
	Kimia hospital	14	3.2
	Antani hospital	19	4.4
	Emamul Mottaqin hospital	29	6.7
	Ghalib hospital	14	3.2
	Others	16	3.7
Total		433	100.0

Knowledge was assessed using 13 items. Overall performance showed a median correct score of 53.8%. In detail, 45.7% of participants (*n* = 198) correctly identified the most important way to control infection in healthcare centers, 26.6% (*n* = 115) recognized the most common type of nosocomial infection, and 40.4% (*n* = 175) correctly identified three types of standard precautions based on transmission routes. Furthermore, 65.6% (*n* = 284) selected the most complete definition of occupational exposure, whereas only 32.6% (*n* = 141) knew the correct arrangement for putting on personal protective equipment. In addition, 44.6% (*n* = 193) identified the ward most important in terms of hospital infection rate, and 51.7% (*n* = 224) correctly indicated which diseases can be prevented by vaccination in hospital settings and the immunity mechanism.

Moreover, 43.4% (*n* = 188) correctly defined nosocomial infections, and 56.6% (*n* = 245) recognized the most important risk factor contributing to increased nosocomial infections. Participants also correctly indicated which patients are most dangerous for dentists. Hepatitis‐B related knowledge was generally better: 85.0% (*n* = 368) identified the most important route of HBV transmission in healthcare workers, 61.9% (*n* = 268) correctly knew the proper timing for HBV vaccination in healthy individuals, and 56.8% (*n* = 246) recognized the minimum required time for rubbing hands following contact with infection‐related sources (Table 2).

**Table 2:** Knowledge of nosocomial infection among health workers (Herat, Afghanistan, 2024).

No.	Knowledge questions	% Correct
1	What is the most important way to control infection in healthcare centers?	45.7
2	What is the most common type of nosocomial infection?	26.6
3	What are Three types of standard precautions based on the way of transmission of infections?	40.4
4	The most complete definition of occupational exposure?	65.6
5	Arrangement of wearing personal protective equipment?	32.6
6	Which ward is more important in terms of hospital infection rate?	44.6
7	Which type of diseases can be prevented in the hospital by vaccination, and how is immunity?	51.7
8	Nosocomial infection definitions?	43.4
9	The most important risk factor for increasing nosocomial infections?	56.6
10	Which are the most dangerous patients for dentists?	56.1
11	The most important way for Hepatitis B infections for healthcare workers?	85.0
12	Timing for getting HBV vaccinations for normal people?	61.9
13	The minimum time for rubbing hands after touching with infections?	56.8
	Overall % correct—median (IQR) = 53.8 (38.4, 61.5)	

Attitude was evaluated using 13 questions. Overall, the median proportion of correct responses was 76.9% (Interquartile Range: 61.5–84.6). In total, 94.7% (*n* = 410) agreed that hand washing is the most important method of infection control in hospitals, and 73.7% (*n* = 319) disagreed with the statement that hand washing is not needed if gloves are worn. Only 5.3% (*n* = 23) stated that gloves are important even when they are assured there is no infection. In addition, 90.1% (*n* = 390) agreed that hand washing is mandatory after contact with a patient's secretions. Furthermore, 71.8% (*n* = 311) agreed that if N95 masks are not available, two masks with two gauzes in four layers must be used to enter isolation rooms. Also, 68.8% (*n* = 298) disagreed that cleaning hospital environments and beds has no role in infection control, and 16.4% (*n* = 71) disagreed that patients with hepatitis B must be isolated.

Among all participants, 91.9% (*n* = 398) agreed that disinfecting patients' instruments has an important role in infection control, 90.3% (*n* = 391) agreed that all staff must receive HBV vaccination before starting work, and 81.3% (*n* = 352) agreed that papers and other items in the ward can transmit infection to patients or staff. In addition, 66.7% (*n* = 289) disagreed that increased workload is not associated with nosocomial infections, 92.8% (*n* = 402) agreed that medical equipment such as NG tubes can cause nosocomial infections, and 94.2% (*n* = 408) agreed that holding training classes and seminars is necessary for staff awareness (Table 3).

**Table 3:** Attitude toward nosocomial infection among health workers (Herat, Afghanistan, 2024).

No.	Attitude questions	% Correct
1	Hand washing is the most important method of infection control in the hospital.	94.7
2	If wearing gloves, there is no need for washing hands.	73.7
3	Despite being assured that there is no infection, wearing gloves is important to protect from being contaminated.	5.3
4	Hand washing is mandatory after contact with the patient's secretions.	90.1
5	If there is no N95 mask, we use two masks with two gauzes in four layers to enter the isolated room.	71.8
6	Cleaning of the hospital environments and patients' beds does not have a role in controlling infections.	68.8
7	Patients with hepatitis B must be isolated.	16.4
8	Disinfections of patients' instruments have a good role in controlling infections.	91.9
9	All staff in the hospital must take HBV vaccinations before starting their work.	90.3
10	Paper and things in the ward can carry infections to staff or patients.	81.3
11	The increasing workload does not have a relation with infection by nosocomial infections.	66.7
12	Most nosocomial infections can be caused by medical equipment like NG‐ tubes…	92.8
13	Classes and Seminars for awareness' of stuff are necessary in hospitals.	94.2
	Overall % correct—median (IQR) = 76.9 (61.5, 84.6)	

Practice was assessed using 13 questions. The median proportion of correct practice was 32.3% (Interquartile Range: 26.1–40.0). In total, 80.8% of participants (*n* = 350) reported that they always wore special uniforms and clothes in the hospital, 66.5% (*n* = 288) stated that they always performed hand hygiene and scrubbing before examining patients, and 68.4% (*n* = 296) reported always observing the correct arrangement for wearing personal protective equipment. In addition, 43.6% (*n* = 189) reported always using glasses or a shield when there was a possibility of splashing during washing activities, and 60.5% (*n* = 262) stated that they always removed rings, watches, and bracelets before washing their hands. Moreover, 49.7% (*n* = 215) reported always participating in classes and seminars related to nosocomial infection control.

Furthermore, 66.1% (n = 286) indicated that they always washed their eyes with plenty of water or normal saline if infected liquids splashed into the eyes, 70.2% (*n* = 304) always disinfected and reported Needlestick injuries, and 73.9% (*n* = 320) always disposed of gloves in infectious waste bins. Additionally, 53.8% (*n* = 233) stated that they always rested at home if they contracted an infectious disease, and 57.3% (*n* = 248) reported always disinfecting their hands with alcohol after contact with patients' papers or equipment. Finally, 50.8% (*n* = 220) reported always wearing a gown in situations where secretion splashing was possible, and 58.0% (*n* = 251) stated that they always did not recap syringes and needles after injecting patients (Table 4).

**Table 4:** Practice toward nosocomial infection among health workers (Herat, Afghanistan, 2024).

No.	Practice questions	Always	Mostly	Sometimes	Rarely	Never
		%	%	%	%	%
1	I wear special Uniforms and clothes in the hospital.	80.8	11.3	6.7	1.2	.0
2	I do hand hygiene and scrubbing before contact or examination of patients.	66.5	21.7	10.2	1.4	.2
3	I observe the Arrangement of wearing personal protective equipment in hospitals.	68.4	17.3	11.3	3.0	.0
4	If there is a possibility of secretions splashing while washing things and the environment, I use glasses or a shield.	43.6	21.9	21.7	9.2	3.5
5	I take off rings, watches, and bracelets before washing my hands.	60.5	15.0	15.9	5.3	3.2
6	I participate in classes and seminars about controlling nosocomial infections.	49.7	18.9	21.2	7.6	2.5
7	In case of splashing infectious liquids in the eye, I wash it completely with plenty of water or normal saline.	66.1	18.7	10.9	3.5	.9
8	In the case of needle sticks, I disinfect my hands with alcohol and report.	70.2	15.5	10.9	2.5	.9
9	After using gloves, I take them out in the infectious bin.	73.9	13.9	9.9	1.4	.9
10	In case of taking infectious diseases, I completely rest at home.	53.8	18.7	18.0	7.2	2.3
11	After contact with the patient's paper and equipment, I disinfected my hand with alcohol.	57.3	23.1	14.5	4.4	.7
12	If there is a possibility of secretions splashing while washing things and the environment, I use glasses or a shield to protect myself.	50.8	22.4	19.6	6.2	.9
13	I do not recap needles and syringes after patients' injections.	58.0	20.1	10.9	6.2	4.8
	Overall % correct—median (IQR) = 32.3 (26.1, 40.0)					

Table 5 shows the univariate analysis of nosocomial infections KAP among HCWS in Herat, Afghanistan. In terms of knowledge, individuals aged 35 years or older had a significantly higher level of knowledge (48.0%) compared with those aged 18–24 years (23.3%) (*p* = 0.009). Married participants showed significantly higher knowledge (36.3%) than single individuals (27.1%) (*p* = 0.040). Those with Master's or Ph.D. degrees demonstrated the highest level of knowledge (54.5%) compared with diploma holders (20.9%) (*p* < 0.001). Medical doctors also exhibited higher knowledge (52.5%) compared with medical technologists (19.4%) (*p* < 0.001). Participants with more than 3 years of experience had higher knowledge (40.5%) than those with three or fewer years of experience (25.4%) (*p* < 0.001). Health workers from private hospitals also had a higher level of knowledge (34.9%) compared to those from Herat Regional Hospital (23.5%) (*p* = 0.024). Overall, 31.9% of healthcare workers demonstrated high knowledge regarding nosocomial infections.

**Table 5:** Univariate analysis of factors associated with good knowledge, attitude, and practice towards nosocomial infection among health workers (Herat, Afghanistan, 2024).

		*N*	High knowledge		*p*‐value	High attitude		*p*‐value	High practice		*p*‐value
			*n*	%		*n*	%		*n*	%	
Age	18‐24	150	35	23.3	0.009	46	30.7	0.366	69	46.0	0.210
	25‐29	155	51	32.9		47	30.3		73	47.1	
	30‐34	78	28	35.9		32	41.0		26	33.3	
	> = 35	50	24	48.0		17	34.0		23	46.0	
Gender	Male	204	65	31.9	0.997	55	27.0	0.015	88	43.1	0.700
	Female	229	73	31.9		87	38.0		103	45.0	
Marital status	Single	207	56	27.1	0.040	58	28.0	0.043	103	49.8	0.024
	Married	226	82	36.3		84	37.2		88	38.9	
Education	Diploma	215	45	20.9	< 0.001	61	28.4	0.037	95	44.2	0.096
	Bachelor & MD	174	69	39.7		60	34.5		83	47.7	
	Master & PhD	44	24	54.5		21	47.7		13	29.5	
Professions	Medical doctor (MD)	101	53	52.5	< 0.001	44	43.6	0.046	43	42.6	0.528
	Nurse	133	33	24.8		34	25.6		65	48.9	
	Midwives	76	27	35.5		25	32.9		35	46.1	
	Medical technologists	67	13	19.4		24	35.8		28	41.8	
	Others	56	12	21.4		15	26.8		20	35.7	
Economic status	Good	107	38	35.5	0.645	35	32.7	0.718	43	40.2	0.641
	Average	200	61	30.5		69	34.5		91	45.5	
	Poor	126	39	31.0		38	30.2		57	45.2	
Employment status	Official	110	46	41.8	0.062	44	40.0	0.104	49	44.5	0.486
	Contract	179	53	29.6		60	33.5		72	40.2	
	Volunteer	105	30	28.6		30	28.6		50	47.6	
	Intern	39	9	23.1		8	20.5		20	51.3	
Work shift	Morning	166	59	35.5	0.411	50	30.1	0.067	79	47.6	0.569
	Evening	111	36	32.4		46	41.4		46	41.4	
	Nightly	58	14	24.1		13	22.4		27	46.6	
	Circulating	98	29	29.6		33	33.7		39	39.8	
Work hours (per day)	8 h or less	390	127	32.6	0.351	122	31.3	0.044	179	45.9	0.024
	More than 8 h	43	11	25.6		20	46.5		12	27.9	
Number of patients visited (per day)	23 patients or less	217	75	34.6	0.228	77	35.5	0.232	86	39.6	0.060
	More than 23 patients	216	63	29.2		65	30.1		105	48.6	
Experience (years)	3 years or less	248	63	25.4	< 0.001	70	28.2	0.019	115	46.4	0.273
	More than 3 years	185	75	40.5		72	38.9		76	41.1	
Health centers	Herat regional hospital	115	27	23.5	0.024	21	18.3	< 0.001	57	49.6	0.169
	Private hospitals		111	34.9		121	38.1		134	42.1	
Total		433	138	31.9		142	32.8		191	44.1	

Regarding attitude, females had significantly higher attitude levels (38.0%) than males (27.0%) (*p* = 0.015). Married individuals showed significantly higher attitude (37.2%) compared to single individuals (28.0%) (*p* = 0.043). Master's and Ph.D. holders had higher attitude (47.7%) compared with diploma holders (28.4%) (*p* = 0.037). Medical doctors had higher attitude (43.6%) than nurses (25.6%) (*p* = 0.046). Individuals working more than 8 h per day showed higher attitude (46.5%) compared to those with 8 h or less (31.3%) (*p* = 0.044). Those with more than 3 years of experience had higher attitude (38.9%) than those with three or fewer years (28.2%) (*p* = 0.019). Individuals from private hospitals had higher attitude (38.1%) compared to HCWs from Herat Regional Hospital (18.3%) (*p* < 0.001). Overall, 32.8% of HCWs demonstrated a high level of attitude toward nosocomial infections.

In terms of practice, single participants demonstrated significantly higher practice (49.8%) compared to married participants (38.9%) (*p* = 0.024). Individuals working 8 h or less per day also showed higher practice (45.9%) compared with those working more than 8 h per day (27.9%) (*p* = 0.024). Overall, 44.1% of HCWs exhibited high practice in preventing nosocomial infections.

Table 6 presents the multivariate analysis of nosocomial infection KAP among HCWs in Herat, Afghanistan. HCWs with a Bachelor's or MD degree were more than twice as likely to demonstrate high knowledge compared with diploma holders (OR = 2.257, 95% CI: 1.261–4.038, *p* = 0.006). Medical doctors were over three times more likely to have high knowledge than the reference group (OR = 3.299, 95% CI: 1.358–8.011, *p* = 0.008), while midwives were also significantly more likely to demonstrate high knowledge (OR = 2.452, 95% CI: 1.001–6.004, *p* = 0.050). Participants with poor economic status were more likely to have high knowledge compared with those with good economic status (OR = 2.250, 95% CI: 1.083–4.675, *p* = 0.030). Volunteers (OR = 2.772, 95% CI: 1.022–7.516, *p* = 0.045) and official staff (OR = 3.138, 95% CI: 1.119–8.798, *p* = 0.030) were also more likely to demonstrate high knowledge compared with interns.

**Table 6:** Multivariate analysis of factors associated with good knowledge, attitude, and practice toward nosocomial infection among health workers (Herat, Afghanistan, 2024).

		Knowledge				Attitude				Practice			
		*p*‐value	OR	95% C.I. for OR		*p*‐value	OR	95% C.I. for OR		*p*‐value	OR	95% C.I. for OR	
				Lower	Upper			Lower	Upper			Lower	Upper
Age	18–24 (Ref)	0.910				0.431				0.402			
	25–29	0.884	1.048	0.561	1.959	0.297	0.725	0.396	1.327	0.664	1.126	0.659	1.924
	30–34	0.705	0.850	0.367	1.972	0.634	0.820	0.362	1.857	0.572	0.803	0.375	1.719
	> = 35	0.844	1.106	0.407	3.004	0.140	0.464	0.167	1.286	0.329	1.580	0.631	3.954
Gender	Male (Ref)												
	Female	0.819	1.063	0.629	1.796	0.005	2.073	1.246	3.449	0.743	1.081	0.678	1.722
Marital status	Single (Ref)												
	Married	0.812	1.067	0.624	1.823	0.097	1.556	0.923	2.625	0.048	0.621	0.387	0.996
Education	Diploma (Ref)	0.022				0.492				0.093			
	Bachelor & MD	0.006	2.257	1.261	4.038	0.657	1.138	0.642	2.017	0.337	1.290	0.767	2.169
	Master & PhD	0.072	2.659	0.917	7.707	0.242	1.911	0.646	5.650	0.232	0.528	0.185	1.505
Professions	Others (Ref)	0.019				0.146				0.531			
	Medical doctor (MD)	0.008	3.299	1.358	8.011	0.058	2.342	0.972	5.642	0.206	1.683	0.751	3.770
	Nurse	0.232	1.648	0.726	3.741	0.857	1.074	0.492	2.347	0.127	1.724	0.857	3.469
	Midwives	0.050	2.452	1.001	6.004	0.927	1.041	0.445	2.435	0.187	1.700	0.772	3.741
	Medical technologists	0.946	0.969	0.389	2.416	0.113	1.956	0.853	4.484	0.524	1.280	0.599	2.734
Economic status	Good (Ref)	0.083				0.076				0.921			
	Average	0.259	1.429	0.769	2.655	0.040	1.891	1.029	3.474	0.722	1.104	0.641	1.902
	Poor	0.030	2.250	1.083	4.675	0.035	2.163	1.057	4.425	0.927	1.030	0.552	1.921
Employment status	Intern (Ref)	0.126				0.245				0.957			
	Official	0.030	3.138	1.119	8.798	0.073	2.535	0.915	7.023	0.955	1.025	0.433	2.428
	Contract	0.119	2.170	0.820	5.743	0.347	1.584	0.607	4.137	0.775	0.889	0.398	1.988
	Volunteer	0.045	2.772	1.022	7.516	0.394	1.539	0.572	4.142	0.795	0.897	0.397	2.029
Work shift	Circulating (Ref)	0.815				0.329				0.913			
	Morning	0.562	1.197	0.652	2.201	0.975	1.010	0.556	1.835	0.527	1.192	0.692	2.054
	Evening	0.854	1.064	0.548	2.066	0.231	1.473	0.782	2.776	0.883	1.046	0.576	1.900
	Nightly	0.678	0.838	0.363	1.932	0.507	0.758	0.335	1.718	0.657	1.175	0.578	2.389
Work hours (per day)	More than 8 h (Ref)												
	8 h or less	0.207	1.668	0.753	3.692	0.104	0.553	0.271	1.129	0.046	2.132	1.014	4.483
Number of patients visited (per day)	More than 23 patients (Ref)												
	23 patients or less	0.734	1.087	0.672	1.760	0.742	0.923	0.574	1.486	0.089	0.686	0.444	1.059
Experience (years)	3 years or less (Ref)												
	More than 3 years	0.144	1.525	0.866	2.685	0.349	1.311	0.744	2.310	0.842	1.053	0.631	1.757
Health centers	Herat regional hospital												
	Private hospitals	0.099	1.666	0.908	3.055	0.003	2.610	1.392	4.891	0.547	0.854	0.510	1.430
Constant		0.000	0.013			0.000	0.049			0.131	0.350		

Regarding attitude, female HCWS were more likely to demonstrate high attitude compared with males (OR = 2.073, 95% CI: 1.246–3.449, *p* = 0.005). Those with average economic status (OR = 1.891, 95% CI: 1.029–3.474, *p* = 0.040) and poor economic status (OR = 2.163, 95% CI: 1.057–4.425, *p* = 0.035) were more likely to demonstrate high attitude than those with good economic status. HCWs in private hospitals were more likely to have high attitude than those in Herat Regional Hospital (OR = 2.610, 95% CI: 1.392–4.891, *p* = 0.003).

In terms of practice, married participants were less likely to demonstrate high practice compared with single participants (OR = 0.621, 95% CI: 0.387–0.996, *p* = 0.048). HCWs who worked 8 h or less per day were more likely to demonstrate high practice than those working more than 8 h per day (OR = 2.132, 95% CI: 1.014–4.483, *p* = 0.046).

## 4 Discussion

Nosocomial infections contribute to prolonged hospitalization, increased mortality, and higher healthcare costs [33]. Preventing and controlling these infections remains a critical global health concern [34]. This study aimed to assess KAP of HCWs in Herat, Afghanistan, regarding nosocomial infection control. Overall, the results indicated a moderate level of knowledge (53.8%), a high level of attitude (76.9%), and a low level of practice (32.3%). Furthermore, a more granular analysis revealed that 31.9% of HCWs demonstrated high knowledge, 32.8% had a high level of attitude, and 44.1% exhibited high practice toward infection prevention. These findings underscore a pronounced knowledge–practice gap, suggesting that even when HCWs possess adequate knowledge and favorable attitudes, this does not consistently translate into optimal implementation of infection control measures within healthcare settings.

### 4.1 Comparison With Other Studies

Compared with HCWs in Saudi Arabia, where over two‐thirds demonstrated good knowledge (67.6%) and strong adherence to infection control practices (73.3%) [34], Afghan HCWs in this study exhibited considerably lower performance across all KAP domains. The superior results in Saudi Arabia likely reflect well‐established infection prevention programs, routine accreditation standards, and regular staff training. Notably, while experience negatively affected knowledge in Saudi Arabia, our data showed the opposite pattern, suggesting that in low‐resource settings such as Afghanistan, practical exposure compensates for limited formal training opportunities.

In Ethiopia, similar knowledge deficits were reported, particularly among younger and less experienced HCWs. As in our study, attitudes were generally positive, yet practice lagged behind, highlighting a common gap between awareness and behavioral implementation in resource‐limited healthcare systems. However, Ethiopian HCWs reported higher overall practice levels, likely due to better institutional enforcement of infection control policies and wider access to refresher training [35].

In China, markedly higher KAP scores were observed, supported by structured infection prevention education, frequent HAI training, and institutional monitoring systems. Predictors of better KAP performance, such as female gender, the nursing profession, and recent educational exposure, were consistent with our findings, although overall practice levels in Afghanistan remained substantially lower, reflecting differences in healthcare infrastructure and ongoing professional development [36].

The Ugandan study also showed moderate knowledge and positive attitudes but low adherence to hand hygiene and PPE use, closely resembling the Afghan context. Workload and insufficient supplies were the main barriers, aligning with our finding that shorter working hours were significantly associated with better practice [37]. Such parallels underscore systemic, rather than individual, obstacles to infection control compliance in low‐income settings.

In the Jamaican study, attitudes were identified as the strongest predictors of infection control practices, supported by access to manuals and continuous training [38]. In our study, HCWs also demonstrated high attitude scores, but practice remained relatively low. This contrast suggests that while positive attitudes are necessary for good practice, they are not sufficient on their own. Effective translation of attitude into practice requires institutional support, training, and resources, which are currently limited in Afghan hospitals.

Across these studies, a consistent pattern emerges: positive attitudes toward infection control are widespread, yet the translation into effective practice varies considerably. In Afghanistan, despite relatively high attitudes (76.9%) and moderate knowledge (53.8%), actual practice remained low (32.3%), highlighting a substantial gap between awareness and implementation. This contrasts with higher‐performing contexts, such as Saudi Arabia, China, and Jamaica, where institutional support, regular training, and resource availability enable attitudes and knowledge to translate more effectively into practice. The Afghan findings underscore that without sustained organizational reinforcement and adequate resources, even motivated and knowledgeable HCWs struggle to consistently implement infection control measures.

### 4.2 Factors Influencing KAP

Our study identified several interrelated factors influencing HCWs' KAP toward nosocomial infection control in Herat, Afghanistan, with professional designation emerging as a key determinant. Physicians demonstrated the highest knowledge and attitudes, consistent with findings from Uganda, where doctors similarly outperformed other cadres in knowledge [37]. Educational attainment also played a critical role: HCWs with Bachelor's and MD outperformed diploma holders, reflecting structured instruction in IPC, enhanced critical thinking, and greater familiarity with evidence‐based practices, consistent with findings from Saudi Arabia and China [34, 36]. Length of service and age positively influenced knowledge and attitudes, likely due to cumulative clinical exposure and experiential learning, echoing observations from Ethiopia [35].

Demographic characteristics, particularly gender and marital status, further shaped KAP. Female HCWs exhibited higher attitude levels, which may reflect greater conscientiousness, attention to detail, or adherence to protocols in the female gender, consistent with findings from China [36]. Marital status influenced practice, with single HCWs demonstrating better adherence to IPC procedures than married counterparts, likely because reduced domestic responsibilities allowed greater focus and energy. This aligns with evidence that work–family conflict can impede proper nursing care, as competing domestic and professional demands may limit adherence to infection‐prevention protocols [39].

HCWs working 8 h or less per day also reported higher practice levels than those on longer shifts, indicating that excessive workload and time pressure undermine consistent IPC implementation. Comparable qualitative evidence from Saudi Arabia similarly shows that high patient load, inadequate staffing, and shift durations exceeding 12 h precipitate fatigue and cognitive depletion, thereby diminishing the capacity to maintain optimal infection‐prevention behaviors [40]. Institutional context mattered as well, with private hospital HCWs showing better KAP, possibly due to more structured supervision, better resource availability, and routine training. These findings align with international studies from Saudi Arabia, China, and Jamaica emphasizing the importance of organizational support, training, and resources in facilitating IPC compliance [34, 36, 38].

To improve KAP comprehensively, interventions must be multi‐faceted and aligned with the determinants identified in this study. Targeted IPC training and mentorship for non‐physician and diploma‐level staff is essential to strengthen procedural knowledge, and less experienced healthcare workers should be paired with senior staff through structured rotations or supervised practice to accelerate skill acquisition. Training must engage both genders equitably, acknowledging that female healthcare workers demonstrated greater receptivity at the attitudinal level. Shift scheduling should prioritize manageable workloads to reduce fatigue‐related lapses in adherence, particularly among married staff and those working extended hours. Institutional support must guarantee stable access to PPE, antiseptics, isolation resources, and routine monitoring, with IPC performance indicators integrated into accreditation and audit frameworks. Regular refreshers, simulation‐based exercises, and participation in infection‐related case discussions should be embedded into continuous professional development. By addressing individual, workload, and institutional factors concurrently, these measures can practically reduce the knowledge–practice gap and enhance patient safety in Afghan healthcare facilities.

### 4.3 Policy Implications

The study highlights a significant knowledge–practice gap among HCWs in Afghanistan, where moderate knowledge (53.8%) and high attitudes (76.9%) did not translate into adequate infection control practices (32.3%). Policies should focus on institutional support and resource provision, including the consistent availability of personal protective equipment, antiseptics, and isolation facilities. Evidence from low‐resource settings such as Uganda and Jamaica indicates that systemic constraints, rather than individual motivation, are major barriers to effective infection control [37, 38]. Ensuring that healthcare facilities are adequately resourced is critical to enable HCWs to implement infection prevention measures consistently.

Structured and ongoing training programs are essential to reinforce infection prevention and control knowledge and attitudes. Studies from China, Saudi Arabia, and Jamaica demonstrate that healthcare workers who receive regular infection control education, antibacterial drug training, and participation in clinical consultations achieve higher knowledge, attitude, and practice scores [34, 36, 38]. In Afghanistan, limited access to refresher courses may explain the low translation of positive attitudes into consistent practices. Policies should mandate routine, hands‐on training for all healthcare staff and encourage mentorship programs where experienced staff support less experienced or non‐clinical personnel.

Workforce management and monitoring systems can further improve adherence to infection control practices. Reduced workload and optimized shift schedules are associated with better compliance with multistep procedures [37, 40]. Institutional monitoring through audits, feedback, and integration of infection prevention indicators into hospital accreditation can sustain improvements in practice. Coordinated policies that combine resource provision, continuous training, workload management, and performance monitoring are necessary to bridge the knowledge–practice gap and strengthen nosocomial infection control in Afghan healthcare facilities.

### 4.4 Strengths and Limitations

This study's strengths include its robust cross sectional design, the use of a well structured and pilot validated questionnaire, and data collection from diverse health facilities in Herat. However, convenience sampling may have introduced selection bias, limiting the generalizability of findings. The self administered questionnaire could also lead to response bias, as HCWs might overreport favorable practices. Additionally, the absence of comprehensive data on the total HCW population in Herat constrains representativeness. Despite these limitations, the study provides valuable empirical insight into infection control KAP among Afghan HCWs and offers direction for targeted interventions.

## Author Contributions

**Mohammad Masudi:** conceptualization, writing – original draft, writing – review and editing, validation, methodology, formal analysis, supervision. **Enayatollah Ejaz:** data curation, investigation, methodology, writing – review and editing. **Mohammad Faisal Wardak:** data curation, investigation, writing – review and editing. **Ali Rahimi:** conceptualization, writing – original draft, writing – review and editing, methodology, formal analysis. **Nasar Ahmad Shayan:** writing – original draft, writing – review and editing, supervision, methodology. **Joseph Christian Obnial:** writing – original draft, writing – review and editing, supervision, methodology. **Don Eliseo Lucero‐Prisno III:** conceptualization, writing – review and editing, supervision.

## Funding

The authors received no specific funding for this work.

## Ethics Statement

The study was approved by the Institutional Review Board of Jami University on November 4, 2023 (Approval No. J.2023.11.4.3). All participants provided written informed consent prior to participating in the study. The confidentiality and privacy of the participants were protected throughout the study, following the Declaration of Helsinki and the ethical principles of research involving human subjects.

## Conflicts of Interest

The authors declare no conflicts of interest.

## Transparency Statement

The lead author Mohammad Masudi affirms that this manuscript is an honest, accurate, and transparent account of the study being reported; that no important aspects of the study have been omitted; and that any discrepancies from the study as planned (and, if relevant, registered) have been explained.

## Data Availability

The datasets generated and/or analyzed during the current study are available from the corresponding author, Dr. Mohammad Masudi (mhmasoudy313@gmail.com), upon reasonable request.
